# Effect of Processing Route on Microstructure and Mechanical Properties in Single-Roll Angular-Rolling

**DOI:** 10.3390/ma13112471

**Published:** 2020-05-28

**Authors:** Hak Hyeon Lee, Kyo Jun Hwang, Hyung Keun Park, Hyoung Seop Kim

**Affiliations:** 1 Department of Materials Science and Engineering, Pohang University of Science and Technology (POSTECH), Pohang 37673, Korea; hakhyeon1005@gmail.com (H.H.L.); hgpark1202@postech.ac.kr (H.K.P.); 2 Graduate Institute of Ferrous Technology, Pohang University of Science and Technology (POSTECH), Pohang 37673, Korea; whang5853@postech.ac.kr; 3 Center for High Entropy Alloy, Pohang University of Science and Technology (POSTECH), Pohang 37673, Korea

**Keywords:** severe plastic deformation, copper, finite element analysis, microstructure design, heterostructures, single-roll angular-rolling

## Abstract

This paper reports the effect of the processing route on the microstructure and mechanical properties in the pure copper sheets processed by single-roll angular-rolling (SRAR). The SRAR process was repeated up to six passes in two processing routes, called routes A and C in equal-channel angular pressing. As the number of passes increased, the heterogeneous evolution of hardness and microstructural heterogeneities between the core and surface regions gradually became intensified in both processing routes. In particular, route A exhibited more prominent partial grain refinement and dislocation localization on the core region than route C. The finite element analysis revealed that the intense microstructural heterogeneities observed in route A were attributed to effective shear strain partitioning between the core and surface regions by the absence of redundant strain. On the other hand, route C induced reverse shearing and cancellation of shear strain over the entire thickness, leading to weak shear strain partitioning and delayed grain refinement. Ultimately, this work suggests that route A is the preferred option to manufacture reverse gradient structures in that the degree of shear strain partitioning and microstructural heterogeneity between the core and surface regions is more efficiently intensified with increasing the number of passes.

## 1. Introduction

Severe plastic deformation (SPD) is an impressive method to construct an ultrafine grained (UFG) structure by imposing extreme levels of shear deformation and hydrostatic pressure in a workpiece. For the last several decades, UFG materials with a grain size of less than 1 μm have demonstrated their unique characteristics in mechanical and functional properties [1,2]. Nevertheless, traditional SPD processes, including high-pressure torsion (HPT), equal-channel angular pressing (ECAP), and accumulate-roll bonding (ARB), have faced two chronic problems in terms of productivity and toughness. The limitation in productivity has been overcome by devising a variety of continuous SPD processes [3,4,5]. However, strengthening by the nontraditional SPD processes still entails an inevitable loss of ductility, leading to insufficient toughness to be utilized as various engineering parts [6,7,8]. Recently, the design of heterogeneous microstructure has been introduced as an emerging scheme to realize outstanding combinations of strength and toughness [9,10]. Heterostructured materials are a new class of materials with artificial microstructural heterogeneities, and their unique strengthening mechanisms effectively contribute to strength–ductility synergy. Accordingly, microstructural approaches using SPD have been shifted to tailoring the heterogeneous microstructure by partial grain refinement, such as plastic flow machining [11] and ultrasonic nanocrystalline surface modification [12,13].

Single-roll angular-rolling (SRAR) is a novel process to break through the above two obstacles of conventional SPD processes. The SRAR process guarantees high productivity in that a rotary roll provides a continuous driving force to move a metallic sheet through specially-designed grooves inside a stationary die [14]. Above all, the SRAR process can concentrate shear strain locally on the core region of the metallic sheet [15]. This strain heterogeneity between the core and surface regions leads to heterogeneous microstructural evolution in recrystallization and grain growth during the subsequent heat treatment. As a consequence, the SRAR process can fabricate a new type of heterostructured material called reverse gradient structure, which has a relatively fine-grained core and coarse-grained surfaces [15]. Furthermore, microstructural heterogeneities of reverse gradient structures can be diversified by manipulating the condition of postannealing after SRAR [16]. The reverse gradient structures boast superior mechanical properties by heterodeformation-induced strengthening mechanisms, surpassing those of conventional homogeneous materials. In other words, the heterostructuring using SRAR originates mainly from latent strain heterogeneity, and the shear strain distribution plays a significant role in determining structural features of heterostructured materials.

Because the SRAR process preserves the original thickness of a workpiece before and after processing, it can flexibly tailor the strain heterogeneity through repetitive passes. In the SRAR process, consecutive passes are able to be conducted with two kinds of processing routes. One is without sample rotation between repetitive passes, and the other is to rotate the metallic sheet 180° with respect to the rolling direction (RD) in every pass. In the conventional ECAP process, these two processing routes are well known as ‘route A’ and ‘route C’, respectively [17]. Furthermore, it has been steadily reported that microstructural evolution and mechanical properties of ECAP-processed materials are strongly dependent on the processing route [18,19,20]. Above all, the SRAR process generates heterogeneous shear strain in the thickness direction of the metallic sheet [14,15], so the processing route should be taken into account as a key parameter in tailoring strain distribution and microstructural heterogeneity. Nevertheless, as far as the authors know, there have been no studies on the processing route in the SRAR process.

This work aims to reveal the effect of the processing route on the microstructure and mechanical properties in the SRAR-processed copper sheet. The SRAR process was repeated up to six passes in routes A and C. The microstructure and mechanical properties were investigated with increasing the number of passes in each route. Although tensile properties of the SRAR-processed materials obeyed the conventional strength-ductility trade-off behavior, the hardness distributions evolved heterogeneously in both processing routes. Furthermore, the materials after the six passes clearly demonstrated microstructural heterogeneities between the core and surface regions, which appeared to be vastly differentiated in routes A and C. The heterogeneities in the two processing routes were clarified based on local evolution of equivalent plastic strain and shear strain, using the finite element method (FEM). Eventually, this work suggests that route A is more beneficial in intensifying strain partitioning and microstructural heterogeneities between the core and surface regions.

## 2. Materials and Methods 

### 2.1. Single-Roll Angular-Rolling

**Figure 1:**
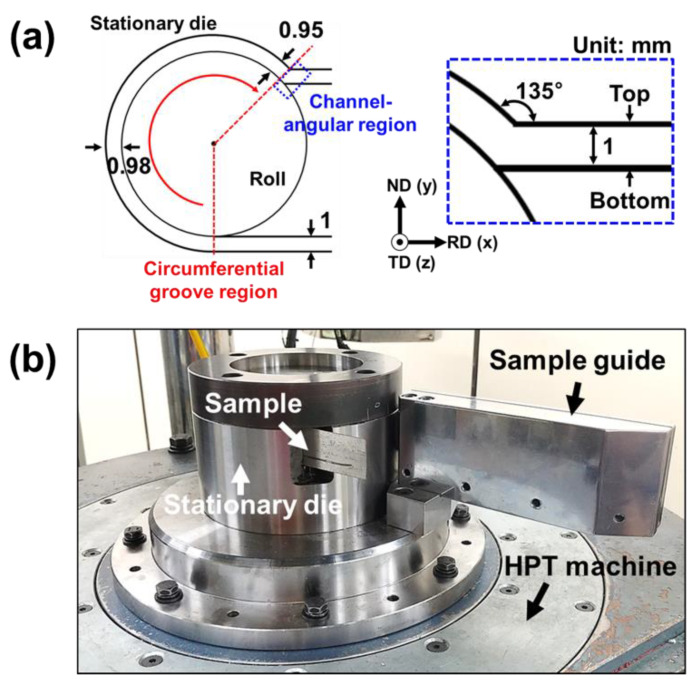
(**a**) Schematic illustration of the single-roll angular-rolling (SRAR) process. (**b**) Experimental setup of the SRAR process customized for the high-pressure torsion (HPT) machine.

In this work, a commercial copper sheet with high purity (99.99%) was employed as a workpiece subjected to the SRAR process. The dimensions of the copper sheet were 300 × 28 × 1 mm^3^, and the sheet was firstly annealed at 600 °C for 2 h to initialize its microstructure. Afterward, the SRAR was carried out at room temperature. The SRAR die used in this study was identical to that in our previous works [14,15,16], whose details are illustrated in Figure 1a. By the friction from the rotary roll, the copper sheet passed through a specially designed groove inside the stationary die. The thickness of the entry groove was designed to be 1 mm, equal to the initial thickness of the copper sheet. The groove thickness gradually decreased to 0.95 mm through the circumferential groove region surrounding the rotating roll. The channel-angular region was devised such that the exit groove intersected the circumferential groove at an angle of 135°. The thickness of the exit groove was designed to be 1 mm to restore the original thickness of the copper sheet by elastic recovery and repulsive force in the channel-angular region. In this work, the SRAR process was customized for the high-pressure torsion (HPT) facility, as shown in Figure 1b. A rotary lower anvil of the HPT machine was used as the roll of the SRAR process, and rotational speed was set to 3 rpm. In addition, a sample guide was installed to prolong the exit groove and alleviate sample deflection by residual stress. The SRAR process was repeated up to six times in two types of processing routes, called ‘route A’ and ‘route C’ in the conventional ECAP process [17]. For convenience, the SRAR-processed samples are referred to in accordance with the number of passes and the processing route (e.g., the sample processed by six passes in route A was named as 6pA). In this work, the number of passes was limited to six passes due to a lack of torque in the HPT machine.

### 2.2. Microstructural Characterization

According to the pass number and the processing route, microstructural evolutions were examined using electron backscatter diffraction (EBSD). After mechanically grinding and polishing to 1200 grit, sample preparation was finalized by electro-chemical polishing using D2 solution. The EBSD measurements were conducted on the plane perpendicular to the transverse direction (TD) plane, using a field-emission scanning electron microscope (FE-SEM, XL-30S FEG, Philips Co., Amsterdam, The Netherlands). An acceleration voltage and a working distance were 25 kV and 12 mm, respectively. Additionally, microstructural morphology on the whole thickness of the SRAR-processed sheets was observed using an optical microscope (OM, BX51RF, Olympus Co., Tokyo, Japan) after chemical etching with 30% nital solution.

To quantify the dislocation density of the SRAR-processed samples, a synchrotron X-ray diffraction (XRD) was implemented using Beamline 8D at the 3.0-GeV Pohang Light Source (PLS) in Korea. This facility employed a double-crystal Si (111) monochromator (POSCO, Pohang, Korea) with a wavelength of 0.151790 nm, and energy resolution (∆*E/E*) was approximately 2 × 10^−4^. The synchrotron XRD was measured on the vertical plane to the normal direction (ND-plane). The XRD patterns were acquired at the range from 30° to 100°. The dislocation density of each material was calculated from the obtained XRD patterns, using the convolution multiple whole profile (CMWP) method [21,22].

### 2.3. Mechanical Testing

Mechanical properties were evaluated using a uniaxial tensile test. A dog-bone shaped sample, with a gauge length of 5 mm and a gauge width of 2.5 mm, was extracted along RD of the SRAR-processed sheet. The tensile test was carried out at a quasi-static strain rate of 1 × 10^−3^ s^−1^, using a universal testing machine (UTM, model 1361, Instron Co., Norwood, MA, USA). To measure highly precise tensile strain, a digital image correlation (DIC, ARAMIS 5M, GOM mbH, Germany) was conducted in parallel with the tensile test, after patterning black and white speckles on the surface of each tensile sample [23]. The sample photographs after the tensile tests are represented in Figure S1a in the Supplementary Material. In addition to the tensile test, Vickers hardness tests (FM-700, Future-Tech Co., Kawasaki, Japan) were performed under a load of 100 gf and with a holding time of 10 s, after mechanically polishing the TD plane to 1200 grit. The hardness values on a specific position were evaluated by averaging at least five measurements.

### 2.4. Finite Element Analysis

To analyze the evolution of local plastic strains in two different processing routes, the 2-D FEM was performed using commercial software, ABAQUS/Standard Ver. 6.9. A deformable part in the FEM simulations was designed as a sheet with the dimensions of 300 (length) × 1 (thickness) mm^2^, identical to the experimental situation. The rotating roll and the stationary die surrounding the roll were realized as discrete rigid bodies, and their geometric details were identical to the experimental ones. The isotropic hardening model using the Swift’s law was applied uniformly over the entire area of the deformable part, as follows:

*σ_p_ = K(ε_p_+ε_0_)^n^*, (1)

where *σ_p_* and *ε_p_* are plastic true stress and strain, respectively. *K*, *n*, and *ε_0_* represent the material strength factor, strain hardening exponent, and strain adjusting factor, respectively, and these parameters were identified as 481.5 MPa, 0.009, and 0.392, respectively, based on the experimental true stress–strain curve of the initially annealed copper. The hardening curve calculated by the Swift’s law was in good agreement with the experimental curve, as shown in Figure S1b in the Supplementary Material. Poisson’s ratio and Young’s modulus were set to be 0.33 and 117 GPa, respectively. The friction coefficients were assumed to be 0.05 on the stationary die as a lubricated condition and 0.6 on the rotary roll with a rough surface [14,15]. A 4-node quadrilateral element under full integration and plane-strain (CPE4) was applied to the workpiece as an element type. The thickness of the workpiece was composed of 15 elements, and the total number of elements was 22,500 for the first pass of the SRAR process. The rotating roll and stationary die consisted of a 2-node linear rigid element, and the number of elements was 800 for the rotating roll and 5000 for the stationary die. The second passes in routes A and C were simulated using a solution mapping technique that numerical solutions of deformed elements after the first pass are transferred to new elements located in the same global coordinate in the second pass [24,25]. This method can efficiently reduce the computational cost of the FEM simulation on SPD processes. The geometry and simulated condition of the second passes were identical to ones of the first pass. The total number of elements constituting the workpiece was set to be 16,500 in both processing routes.

## 3. Results and Discussion

### 3.1. Microstructural Characteristics in the Core Region 

**Figure 2:**
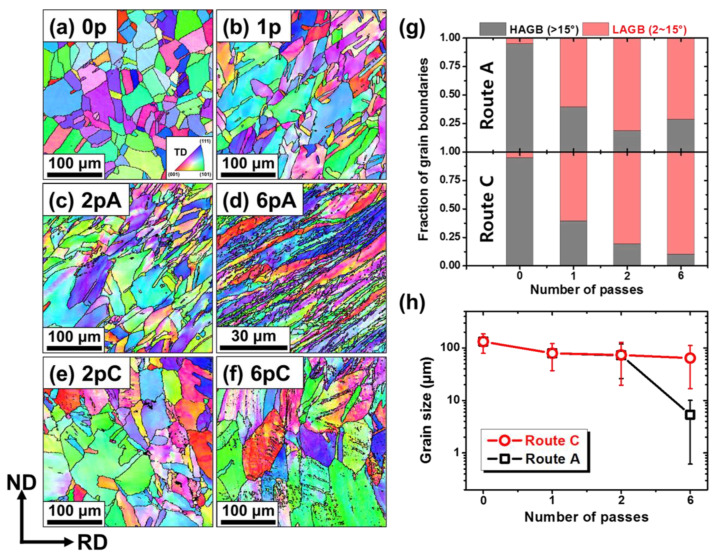
Inverse pole figure (IPF) maps on the core region of the (**a**) as-annealed and (**b**–**f**) SRAR-processed copper sheets. (**g**) Fraction of grain boundaries and (**h**) grain size variations according to the pass number in routes A and C.

The microstructural features of the SRAR-processed sheets were investigated preferentially upon the core region, as represented in Figure 2. The as-annealed sample (0p) subjected to the initial heat treatment exhibited the homogeneous microstructure with equiaxed grains, as shown in Figure 2a. An average grain size of the as-annealed sample was approximately 132.5 μm. It should be noted that twin boundaries were not regarded as grain boundaries in grain size calculations. After one pass of the SRAR process, in-grain misorientations were observed, and the grain morphology was slightly inclined to RD. As the number of passes increased, the misorientation and grain inclination became further intensified in route A, as shown in Figure 2b–d. This implies that the shear deformation in route A continued to be accumulated with the number of passes. On the other hand, Figure 2e,f demonstrate that the grain morphology in route C was restored to nearly equiaxed shape in even-numbered passes even though in-grain misorientations gradually became severe. This indicates that the even-numbered process in route C canceled out most of the shear deformation induced in the previous odd-numbered process. The microstructural appearance observed in the SRAR process was well consistent with that in conventional ECAP processes [26,27]. This similarity in two processes is attributable to the fact that the deformation behavior in the core region of the SRAR-processed materials is primarily determined by simple shear at the channel-angular region [14,15]. Accordingly, the SRAR process in route A continues to intensify shear strain by the next shear plane intersecting at 45° in every repetitive pass, similar to that of the ECAP process with the channel angle of 135° [28]. On the contrary, route C completely compensates for the shear strain induced in the previous pass because only the shear direction is reversed on the identical shear plane.

Furthermore, the different shear behaviors of routes A and C varied the strain level at which grain refinement was activated. Figure 2g demonstrates the variation of grain boundary misorientation according to the number of passes and the processing route. When a misorientation between neighboring pixels in the EBSD results was between 2° and 15°, the boundary of adjacent pixels was regarded as a low-angle grain boundary (LAGB), while the boundary with misorientation angle larger than 15° was considered as a high-angle grain boundary (HAGB). The results on the grain boundary misorientation of each material are represented in Figure S2 and Supplementary Material. For fractions of grain boundaries in Figure 2g, routes A and C exhibited a common tendency that the fraction of low-angle grain boundaries (LAGBs) dramatically increased with increasing the number of passes. However, in the sixth passes, high-angle grain boundaries (HAGBs) began to rise in route A, while a declining tendency remained in route C. In general, dislocations are explosively generated at the beginning of SPD processes, which results in plenty of dislocation cells and intragranular substructures with LAGBs [29,30]. Increasing the applied plastic deformation resulted in more pileups of dislocations aggravating misorientations of the subgrain boundaries. Ultimately, grain refinement and the corresponding increase of HAGBs are initiated by dynamic recovery through dislocation annihilation [19,30]. In other words, the increase in the fraction of HAGBs is the direct evidence that grain refinement is initiated. In fact, the grain size variations in Figure 2h demonstrate that the grain refinement was preferentially activated in route A rather than in route C, despite the same number of passes. This discrepancy was supposed to be due to redundant strain by reverse shearing in route C. The reverse shear strain on the same shear plane can interfere with the accumulation of dislocation and grain refinement by partially reversing dislocation motion [31,32]. The phenomenon becomes more pronounced in ECAP-based processes with an obtuse channel angle than with an acute angle [28]. This SRAR process with the channel angle of 135° generated a shear strain of approximately 0.6 per pass [15], which would be a strain level low enough to cause partial dislocation restoration [33]. Therefore, the grain refinement in the SRAR process was more effective in route A without redundant strain than in route C.

Additionally, the inclination of grains toward RD appeared to be relatively slight in the SRAR process compared to the theoretical inclination in the ECAP process. In the ECAP process, a theoretical shear strain (*γ*) can be calculated depending on the channel angle (*φ*), as follows [34]:

γ = 2cot(*φ*/2). (2)

Based on Equation (2), the theoretical shear strain of the ECAP process with the channel angle of 135° was approximately 0.828 for a single pass, and the corresponding inclination angle with respect to the RD axis was about 50.4°. However, the inclination angle after the first pass of SRAR was approximately 59.9°. In other words, the shear strain by the SRAR process was weaker than that by the ECAP process with the same channel angle. This was primarily due to preferential shear deformation at the circumferential groove region before the workpiece reached the channel-angular region. The pre-deformation called circumferential shear deformation (CSD) was the shear deformation caused by the radial plane torsion of the rotational axis of roll [14]. Although the CSD was much weaker than the channel-angular shear deformation (CASD), it induced a negative shear strain (ε_xy_ < 0) as opposite to that by CASD [15]. Therefore, the CSD canceled out a part of the CASD, which allowed the grain morphology to appear less inclined. Consequently, the microstructural variations on the core region in the SRAR process exhibited parallel results with the conventional ECAP process, except for the slight cancellation of shear strain.

### 3.2. Mechanical Properties

**Figure 3:**
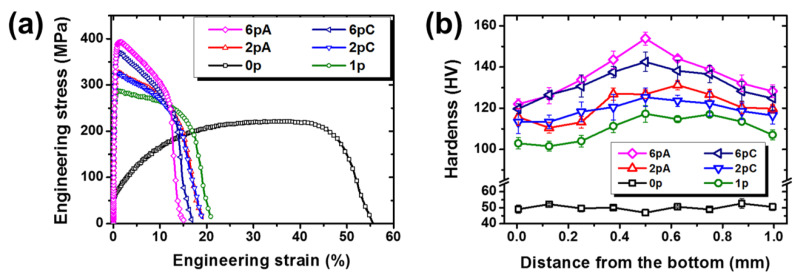
(**a**) Tensile stress–strain curves and (**b**) Vickers hardness distributions along the thickness, depending on the number of passes and the processing route.

The mechanical properties according to the pass number and the processing route were evaluated using tensile stress-strain curves in Figure 3a. The as-annealed sample in the strain-free state had a low yield strength, but the significant strengthening was observed after the first pass of the SRAR process. With further passes, both yield strength and ultimate tensile strength were gradually enhanced by work hardening, as summarized in Table 1. On the other hand, the ductility tended to be opposite to tensile strength. With increasing the number of passes, the elongation declined sharply due to saturated strain hardening ability, leading to a conventional strength-ductility trade-off tendency [35]. As for the processing route, there were no significant differences in tensile properties between routes A and C until two passes. However, the 6pA sample manifested obvious superiority in tensile strength over that of the 6pC sample. Taking the microstructural features of Figure 2 into account, the further strengthening observed in six passes of route A was inferred to be due to the grain-boundary strengthening by preferential grain refinement.

Although the tensile properties of the SRAR-processed materials were under the normal trend of the SPD-processed materials, the SRAR process revealed a uniqueness in that it induced heterogeneous hardening depending on the thickness position. Figure 3b demonstrates the heterogeneous evolution of hardness by the SRAR process. Firstly, the as-annealed sample exhibited a uniform hardness distribution throughout the thickness, from 49.1 to 52.7 HV. While the first pass of the SRAR led to the significant hardening throughout the thickness, the degree of hardening was relatively weak in the vicinity of both surfaces, especially in the bottom region. As increasing the number of passes, the hardness partition between the core and surface regions gradually became intensified in both routes A and C. In particular, the 6pA sample had even higher hardness in the core region than the 6pC sample and represented the prominent hardness heterogeneity. Additionally, the hardening in route C seemed to evolve more symmetrically compared to route A. This was due to the sample rotation of 180° by which the bottom surface in the previous pass became the top surface in the next pass. Consequently, the hardness distributions in Figure 3b imply that the repetition of the SRAR process amplified the intrinsic hardness heterogeneity of the workpiece itself, which was more pronounced in route A than in route C.

### 3.3. Microstructural Heterogeneities in Routes A and C

**Figure 4:**
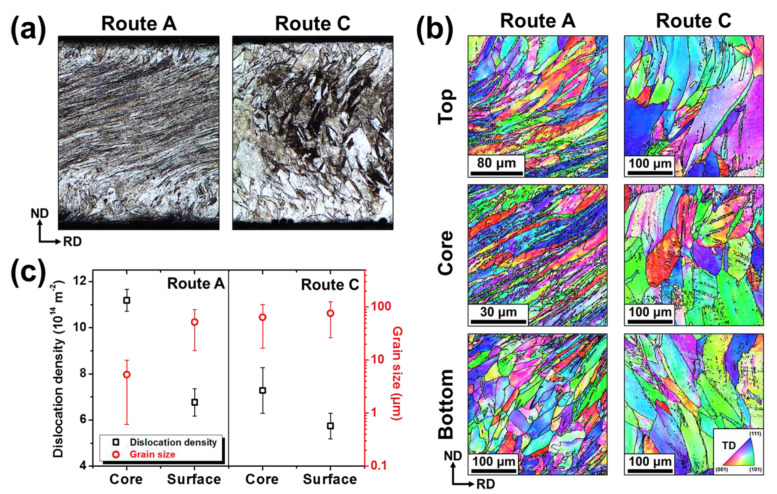
Microstructural heterogeneities of the 6pA and 6pC samples: (**a**) Microstructural morphologies on the whole transverse direction (TD) plane. (**b**) IPF maps on the bottom, core, and top regions. (**c**) Dislocation densities and grain sizes on the core and surface regions.

The hardness heterogeneities observed in SRAR-processed materials were closely associated with the heterogeneous evolution of microstructure depending on the thickness position. Microstructures on the whole thickness of the 6pA and 6pC samples were investigated, as shown in Figure 4a,b, and they revealed distinguished microstructural heterogeneities in routes A and C. For the 6pA sample, the severely distorted microstructures and partial grain refinement were observed in the core region, while both surfaces consist of much less sheared coarse grains. The less sheared zone (LSZ) was formed wider in the vicinity of the bottom surface than the top surface. On the other hand, both surfaces of the 6pC sample exhibited slightly inclined microstructures with coarse grains, in contrast to the core region where nearly equiaxed microstructure was observed by complete reverse shearing and shape restoration. Furthermore, the shear deformation experienced by both surfaces of the 6pC sample appeared to be in the opposite direction. In Figure 4c, dislocation densities and grain sizes of the 6pA and 6pC samples were quantified separately on the core and surface regions. On the whole, the accumulation of dislocations was more substantial in route A than in route C, which was consistent with the results of the ECAP process [18,28,36]. The relatively low dislocation densities in route C were inferred to be due to partial dislocation restoration and rearrangement by reverse shearing. Above all, the core region of the 6pA sample had much higher dislocation densities and more prominent grain refinement than the surface region. On the other hand, the 6pC sample also had higher dislocation densities in the core region similar to the 6pA sample, but the dislocation heterogeneity between the core and surface regions was relatively slight compared to that of the 6pA sample. Besides, there was little difference in the grain size between the core and surface regions in the 6pC sample. In other words, these results demonstrated that route A promoted microstructural heterogeneities between the core and surface regions, compared to route C.

**Figure 5:**
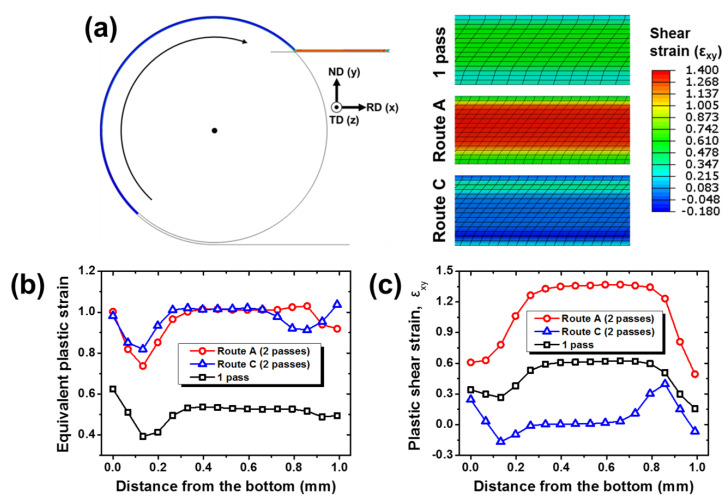
Finite element analysis on the first and second passes in routes A and C of the SRAR process: (**a**) shear strain contour maps, (**b**) equivalent strain distributions, and (**c**) shear strain distributions in the thickness direction.

The mechanical and microstructural heterogeneities in routes A and C were interpreted in terms of equivalent plastic strain and shear strain, using the finite element analysis, as shown in Figure 5. In the first pass, the core region involved uniformly distributed equivalent strains, as represented in Figure 5b. However, both surface regions contained relatively low equivalent strain, especially near the bottom region. This heterogeneous equivalent strain distribution calculated by FEM was in good agreement with the experimental trend of hardness in Figure 3b. Above all, the shear strain distribution after the first pass in Figure 5c proved that the SRAR process dramatically split the level of shear strain between the core and surface regions. The heterogeneous shear distribution resulted from the superposition of three types of shear deformation: CSD, CASD, and frictional shear deformation [15]. The CSD at the circumferential groove region concentrates negative shear strain (ε_xy_ < 0), mainly on the bottom surface of the workpiece. The CASD is the same deformation as the simple shear in the conventional ECAP process in that it derives a substantial amount of positive shear strain (ε_xy_ > 0) uniformly over the entire thickness under an ideal condition. However, the friction between the workpiece and the stationary die induces additional negative shear strain (ε_xy_ < 0) in the vicinity of the top surface during CASD [37]. Accordingly, the CSD and the frictional effect alleviate the positive shear strain by CASD on both surfaces, leading to the shear strain partitioning between the core and surface regions after a single pass of the SRAR process.

The heterogeneous shear strain in SRAR appeared to evolve quite differently in two processing routes. Regarding the equivalent plastic strain of Figure 5b, the second pass in route A without sample rotation between passes increased only the magnitude of equivalent strain while maintaining the qualitative distribution of the first pass. Therefore, the gap of equivalent strain between the core and surface regions became further expanded. For the second pass in route C, the equivalent strain division between the core and surface regions was also intensified, but the less deformed regions expanded even wider on both surfaces than those in route A. Furthermore, the equivalent strain distribution developed more symmetrically in route C. This is because the bottom region, which had undergone much lower deformation in the first pass, became the top region of the second pass in route C. These equivalent strain distributions in routes A and C calculated using FEM showed good consistency with the hardness results in Figure 3b. Above all, the second passes in routes A and C displayed a remarkable distinction in shear strain distributions of Figure 5c. The second pass in route A concentrated the considerable level of shear strain to the core region rather than both surfaces, by doubling the magnitude of shear strain over the entire thickness. On the contrary, the second pass in route C had little shear strain in the core region by completely canceling out the shear strain induced in the first pass, but rather the shear strain was accumulated on both surfaces. Besides, the shear strains on both surfaces were activated in the opposite direction, and their magnitude was significantly feeble compared to that in route A. The shear accumulation on the surfaces in route C was supposed to be due to the fact that a single pass of SRAR generated asymmetric shear strain distribution with a wider LSZ on the bottom region. In summary, both processing routes had similar equivalent strain distribution; however, the shear strain partitioning developed more effectively in route A without redundant shear strain than in route C. This led to further prominent microstructure heterogeneities in route A, such as partial grain refinement and dislocation localization. Additionally, the shear strain in this FEM simulation was calculated based on the shear displacement of elements before and after deformation, so that it can be correlated with the microstructural inclination. Additionally, it is clear that the shear strain partitioning in both processing routes will become further intensified with an increasing number of passes. Therefore, the shear strain distributions in Figure 5c were regarded to be in good agreement with the microstructural morphology observed in Figure 4a,b.

This work demonstrated that route A can effectively promote the shear strain heterogeneity and dislocation localization on the core region, suggesting that route A is more favorable for designing a gradient structure than route C. Indeed, the 6pA sample had grain arrangement of a reverse gradient structure in which the grain size gradually decreased toward the core region by partial grain refinement (Figure 4). Nevertheless, its mechanical properties in Figure 3a manifested little strength–ductility synergy often reported in heterostructured materials [10,38,39,40]. This was because the strain hardening capability had already saturated by a significant amount of plastic work over the entire thickness. However, the depleted strain hardening of the 6pA sample can be restored by reconstructing the deformed microstructure into a sound gradient structure by the postannealing process. As represented in Figure 4c, the 6pA sample had even higher dislocation density on the core region by intense shear strain partitioning. The higher deformation energy stored in the core region can activate more nucleation site and grain boundary impingement in recrystallization and grain growth, leading to the local evolution of fine-grained microstructures [41]. Accordingly, the postannealing process after SRAR can release internal dislocations while maintaining the reverse gradient structure to some extent, which has been demonstrated in our previous reports [15,16]. In other words, the heterostructuring in the SRAR process strongly depends on the degree of shear strain partitioning and dislocation heterogeneity between the core and surface regions. Therefore, route A is the more advantageous option to fabricate reverse gradient structures than route C with relatively weak heterogeneities caused by redundant strains.

## 4. Conclusions

In this study, the influences of routes A and C on the microstructure and mechanical properties were investigated in the SRAR-processed copper sheet. The microstructural and mechanical variations were experimentally examined by increasing the number of passes. Depending on the pass number and the processing route, the microstructural features on the core region and the tensile properties were quite similar to the results in conventional ECAP processes. However, the SRAR process exhibited a uniqueness in that hardness distribution and microstructure heterogeneously developed in the thickness direction. In both processing routes, the SRAR process intensively hardened the core region rather than the surface region, and the hardness heterogeneities were further intensified as the number of passes increased. However, the 6pA sample exhibited more prominent hardness heterogeneity between the core and surfaces than the 6pC sample. This was primarily attributed to the partial grain refinement and dislocation localization on the core region in the 6pA sample. On the other hand, the 6pC sample had no grain refinement, and the dislocation heterogeneity between the core and surface regions was significantly feeble compared to that of the 6pA sample. The finite element analysis revealed that intense microstructural heterogeneities in route A were mainly due to effective shear strain partitioning by the absence of redundant shear strain. Ultimately, this work suggests that the fabrication of reverse gradient structures prefers route A which can effectively boost the degree of the shear strain partitioning and dislocation heterogeneity.

## Figures and Tables

**Table 1:** Tensile properties according to the pass number and the processing route of SRAR.

Samples	YS, MPa	UTS, MPa	U.E., %	T.E., %
0p	58.0 ± 1.9	217.6 ± 7.1	34.7 ± 0.3	54.7 ± 1.8
1p	268.9 ± 7.3	284.9 ± 8.3	1.3 ± 0.1	22.2 ± 1.3
2pA	315.0 ± 0.8	327.8 ± 1.5	1.2 ± 0.1	20.1 ± 0.2
2pC	311.3 ± 1.6	325.6 ± 0.7	0.9 ± 0.1	20.9 ± 1.4
6pA	352.1 ± 3.0	389.9 ± 3.8	1.5 ± 0.2	16.6 ± 0.5
6pC	336.6 ± 1.6	366.6 ± 2.5	1.1 ± 0.1	17.6 ± 1.0

^1^ YS, UTS, U.E., and T.E. represent the yield strength, ultimate tensile strength, uniform elongation, and total elongation, respectively.

## Supplementary Materials

The following are available online at https://www.mdpi.com/1996-1944/13/11/2471/s1, Figure S1: (a) Photographs for the tensile samples, (b) The plastic true stress-strain curves fitted by the Swift’s hardening model. The inset is a photograph of the as-annealed tensile specimen, Figure S2: Grain boundary misorientation maps in the core region of the (a) as-annealed and (b–f) SRAR-processed copper sheets, Table S1: Grain boundary fractions according to the number of passes and processing routes.
