# Supplementary material for: Effect of Processing Route on Microstructure and Mechanical Properties in Single-Roll Angular-Rolling
Source: Materials (Basel). 2020 May 28;13(11):2471. doi: 10.3390/ma13112471 (PMC7321117; doi:10.3390/ma13112471)
Supplement: Supplementary file 1 [file materials-13-02471-s001.pdf]

materials

Supplementary Material

Effect of Processing Route on Microstructure and Mechanical Properties in Single-Roll Angular-Rolling

Hak Hyeon Lee [1], Kyo Jun Hwang [2], Hyung Keun Park [1] and Hyoung Seop Kim [1,2,3,*]

1 Department of Materials Science and Engineering, Pohang University of Science and Technology (POSTECH), Pohang 37673, Korea; hakhyeon1005@gmail.com (H.H.L.); hgpark1202@postech.ac.kr (H.K.P.);
2 Graduate Institute of Ferrous Technology, Pohang University of Science and Technology (POSTECH), Pohang 37673, Korea; whang5853@postech.ac.kr
3 Center for High Entropy Alloy, Pohang University of Science and Technology (POSTECH), Pohang 37673, Korea;
* Correspondence: hskim@postech.ac.kr; Tel.: +82-54-279-2150

Received: 26 April 2020; Accepted: 27 May 2020; Published: date

[Tensile sample and isotropic hardening model used in the FEM]

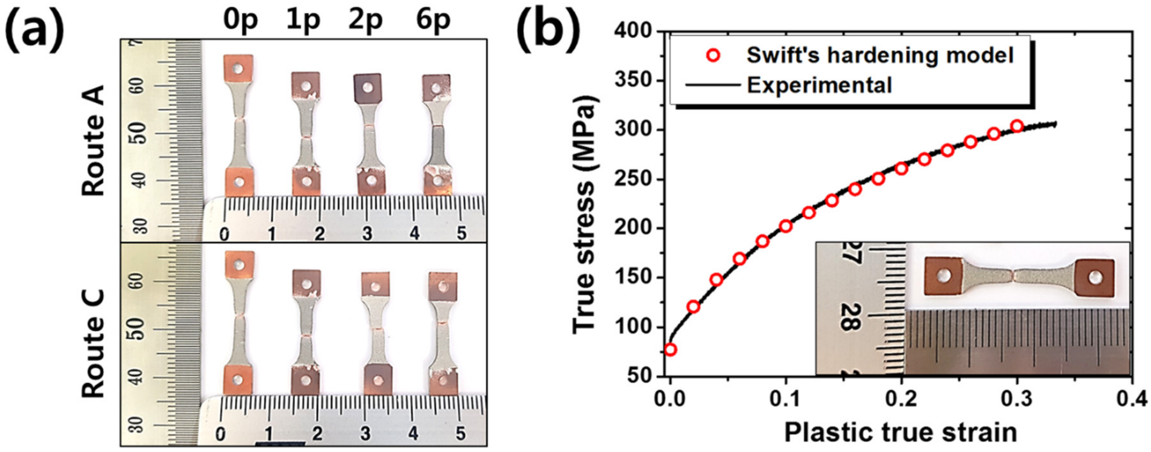

Figure S1. (**a**) Photographs for the tensile samples, (**b**) The plastic true stress-strain curves fitted by the Swift's hardening model. The inset is a photograph of the as-annealed tensile specimen.

[Grain boundary misorientation map]

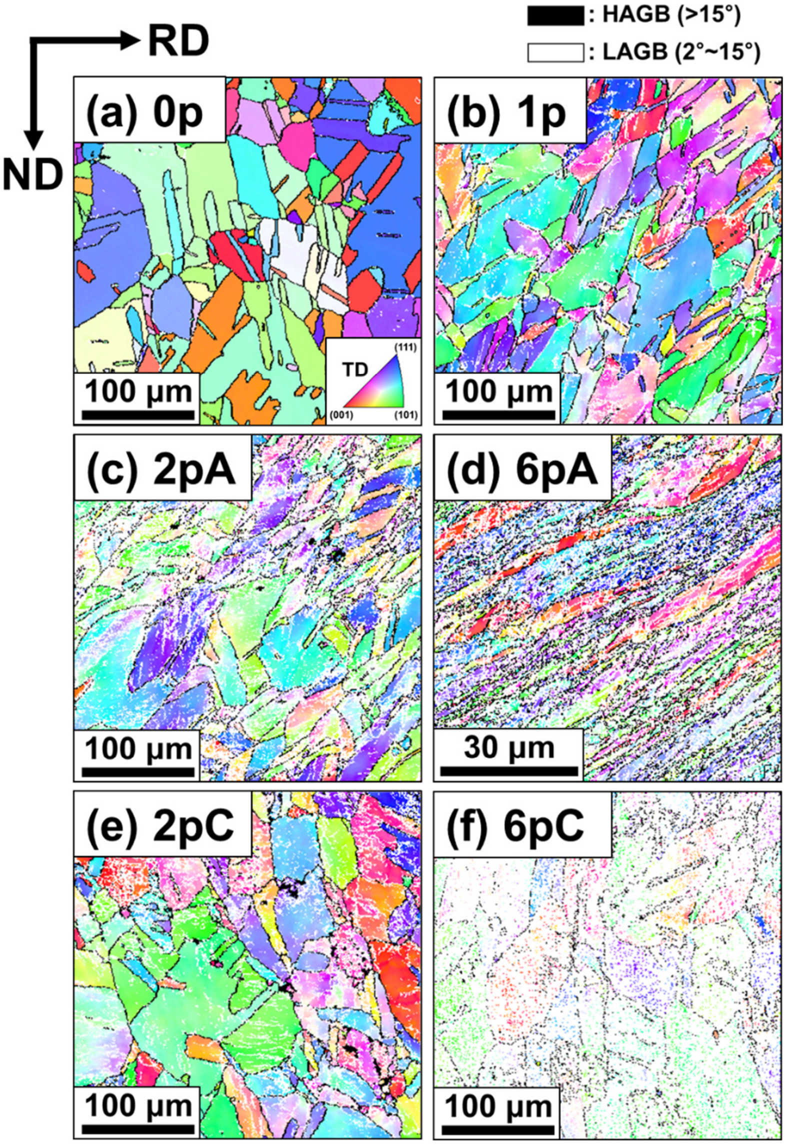

Figure S2. Grain boundary misorientation maps in the core region of the (**a**) as-annealed and (**b**–**f**) SRAR-processed copper sheets.

Table S1. Grain boundary fractions according to the number of passes and processing routes.

Samples	0p	1p	2pA	6pA	2pC	6pC
Fraction of HAGBs	0.948	0.393	0.184	0.285	0.190	0.101
Fraction of LAGBs	0.052	0.607	0.816	0.715	0.810	0.899

© 2019 by the authors. Submitted for possible open access publication under the terms and conditions of the Creative Commons Attribution (CC BY) license (http://creativecommons.org/licenses/by/4.0/).
